# University of Louisville

**Published:** 1854-01

**Authors:** 


### UNIVERSITY OF LOUISVILLE.

We are sure it will be gratifying to many of our readers to learn that this Institution is in a flourishing condition. The Law and Medical Departments, the only two yet in operation, have large classes. The number of students in the latter is greater than it has been for four years, and in the former there has never been, we believe, a larger class. The quiet and gentlemanly deportment of the students in both departments is a subject of general remark with the citizens of Louisville. They are students indeed, and not merely in name, and are making the most of their opportunities for professional improvement.

Professor Flint expects to sail for Europe immediately after the close of the present session, and will purchase for the Medical Department additional means for illustration in the several branches of Medicine.

